# Insights into Electrocatalyst Transformations Studied in Real Time with Electrochemical Liquid-Phase Transmission Electron Microscopy

**DOI:** 10.1021/acs.accounts.3c00463

**Published:** 2023-10-24

**Authors:** Tzu-Hsien Shen, Robin Girod, Vasiliki Tileli

**Affiliations:** Institute of Materials, École Polytechnique Fédérale de Lausanne, CH-1015 Lausanne, Switzerland

## Abstract

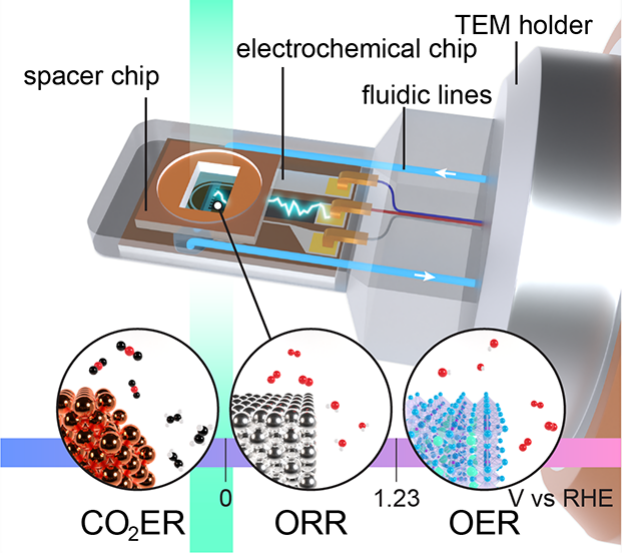

The value of operando and in situ characterization methodologies for understanding electrochemical systems under operation can be inferred from the upsurge of studies that have reported mechanistic insights into electrocatalytic processes based on such measurements. Despite the widespread availability of performing dynamic experiments nowadays, these techniques are in their infancy because the complexity of the experimental design and the collection and analysis of data remain challenging, effectively necessitating future developments. It is also due to their extensive use that a dedicated *modus operandi* for acquiring dynamic electrocatalytic information is imperative. In this Account, we focus on the work of our laboratory on electrochemical liquid-phase transmission electron microscopy (ec-LPTEM) to understand the transformation/activation of state-of-the-art nanocatalysts for the oxygen evolution reaction (OER), oxygen reduction reaction (ORR), and CO_2_ electroreduction (CO_2_ER). We begin by describing the development of electrochemical microcells for TEM studies, highlighting the importance of tailoring the system to each electrochemical process to obtain reliable results. Starting with the anodic OER for alkaline electrolyzers, we demonstrate the capability of real-time monitoring of the electrowetting behavior of Co-based oxide catalysts and detail the fascinating insights gained into solid–liquid interfaces for the reversible surface reconstruction of the catalystic surfaces and their degradation processes. Importantly, in the case of the OER, we report the exceptional capacity of ec-LPTEM to probe gaseous products and therefore resolve solid–liquid–gas phenomena. Moving toward the cathodic ORR for fuel cells, we summarize studies that pertain to the evaluation of the degradation mechanisms of Pt nanoparticles and discuss the issues with performing real-time measurements on realistic catalyst layers that are composed of the carbon support, ionomer network, and Pt nanocatalysts. For the most cathodic CO_2_ER, we first discuss the challenges of spatiotemporal data collection in microcells under these negative potentials. We then show that control over the electrochemical stimuli is critical for determining the mechanism of restructuring/dissolution of Cu nanospheres, either for focusing on the first stages of the reaction or for start/stop operation studies. Finally, we close this Account with the possible evolution in the way we visualize electrochemical processes with ec-LPTEM and emphasize the need for studies that bridge the scales with the ultimate goal of fully evaluating the impact of the insights obtained from the in situ-monitored processes on the operability of electrocatalytic devices.

## Key References

- ShenT. H.; GirodR.; VavraJ.; TileliV.Considerations of Liquid-Phase Transmission Electron Microscopy Applied to Heterogeneous Electrocatalysis. J. Electrochem. Soc.2023, 170, 05650210.1149/1945-7111/acced4.^1^ In this contribution, we detailed guidelines for the experimental design of electrochemical measurements in TEM liquid microcells. By implementing these strageties, we demonstrated facet-dependent product detection and dissolution processes during aging and start/stop operation for the electrocatalytic processes discussed herein.
- ShenT.-H.; SpillaneL.; PengJ.; Shao-HornY.; TileliV.Switchable Wetting of Oxygen-Evolving Oxide Catalysts. Nat. Catal.2022, 5, 30–3610.1038/s41929-021-00723-w35141468PMC8799463.^2^ In this breakthrough study, we reported on potential-regulated and reversible wetting behavior in alkaline OER oxide catalysts that was directly related to changes in the solid–liquid interfacial capacitance through surface redox reactions and electrocatalytic product formation.
- VavraJ.; ShenT. H.; StoianD.; TileliV.; BuonsantiR.Real-time Monitoring Reveals Dissolution/Redeposition Mechanism in Copper Nanocatalysts during the Initial Stages of the CO_2_ Reduction Reaction. Angew. Chem., Int. Ed.2021, 60, 1347–135410.1002/anie.20201113732997884.^3^ In this work, we reported on a customized electrochemical liquid cell TEM configuration that enabled us to elucidate for the first time the activation mechanism of copper nanospheres during open circuit potential, start up, and operation under realistic CO_2_ electroreduction conditions.

## Introduction

An understanding of energy conversion processes is critical to net-zero energy devices toward fossil-fuel-free transportation and industrial chemicals production.^4^ To meet the projected needs at scale, electrocatalysts are used to accelerate the kinetics of the electrochemical reactions.^5^ In most cases, however, and despite advancements, current catalysts lack the durability and selectivity required for the industrial application of these technologies. Toward the goal of highly stable catalysts during electrochemical reactions, in situ and operando characterization techniques are valuable tools. They offer access to real-time changes during reaction and a window on the transient state of the catalysts and on their fundamental working and degradation mechanisms.^6^ Numerous characterization techniques can provide such insights, yet direct visualization of these processes can be achieved largely through in situ transmission electron microscopy (TEM) methodologies.

In particular, the invention of closed cells as a mean to fully isolate their content from the TEM column was pivotal for monitoring electrocatalytic processes in situ because it enabled the observation of samples in liquids in the so-called liquid-phase (LP) TEM.^7,8^ The first closed cells were made from simple plastic films deposited onto centrally bored Pt disks,^9^ but with the advent of modern microelectromechanical systems (MEMS) fabrication techniques, cells are now made by stacking two silicon chips that feature electron-transparent silicon nitride (SiN_*x*_) membranes.^10^ In this configuration, one MEMS chip, the so-called spacer, features pads that create a channel opening (100 nm–2 μm in height) running across the windows, while the other has electrodes patterned on it. The first cells of this kind were fully isolated; that is, liquid was drop cast before stacking the chips or manually injected via dedicated ports in one of the chips. The ports and chips were subsequently sealed with glue, while electrical contact was made with a potentiostat in a two- or three-electrode configuration. This first electrochemical (ec-)LPTEM apparatus enabled a seminal study of copper electrodeposition.^10^ Since then, commercial, precision-machined holders have become widely available. Although various configurations now exist, they have in common that sealing between the chips and the body of the holder is made by O-rings and that fluidic lines run through the shaft of the holder and enable the electrolyte to flow through the cells. This setup enabled ec-LPTEM to be increasingly used in the context of electrocatalysis. In parallel, the advent of direct electron detection cameras with fast readout and high-speed recording improved the TEM imaging capabilities by allowing the acquisition of data with ultralow electron dose rates that limit electron-beam-induced radiolysis effects when combined with thin liquid conditions.^1^ We and others have been active in developing and implementing this setup for a range of electrocatalytic systems,^1^ and this has allowed us to probe solid–liquid interfaces and the morphological evolution of nanocatalysts^3^ and to move toward product detection with spectroscopy.^2,11^

In this Account, we review the use of ec-LPTEM for the study of electrocatalysts. We first present considerations of specific features pertaining to the ec-LPTEM system, including cell geometry and limitations of scale and materials. Then, we discuss studies on the tranformations of electrocatalysts under biasing in liquid electrolytes for systems including the oxygen evolution reaction (OER), oxygen reduction reaction (ORR), and CO_2_ electroreduction (CO_2_ER). For each of these cases, we highlight parameters of importance for ec-LPTEM studies and discuss insights gained from observing catalyst evolution in real-time. Finally, we conclude by offering a perspective on emerging and future applications of ec-LPTEM along with challenges associated with studying the transformations of electrocatalysts in the TEM.

## Electrocatalytic Measurements in TEM Microcells

By design, monitoring electrocatalytic processes via ec-LPTEM measurements deviates from the conditions found in bulk ones, including rotating disk electrodes or H-cells. Thus, we discuss the considerations of the apparatus toward its proper electrochemical operation, which are inherently decoupled from possible sample- and liquid-specific electron-beam-induced effects.

**Figure 1:**
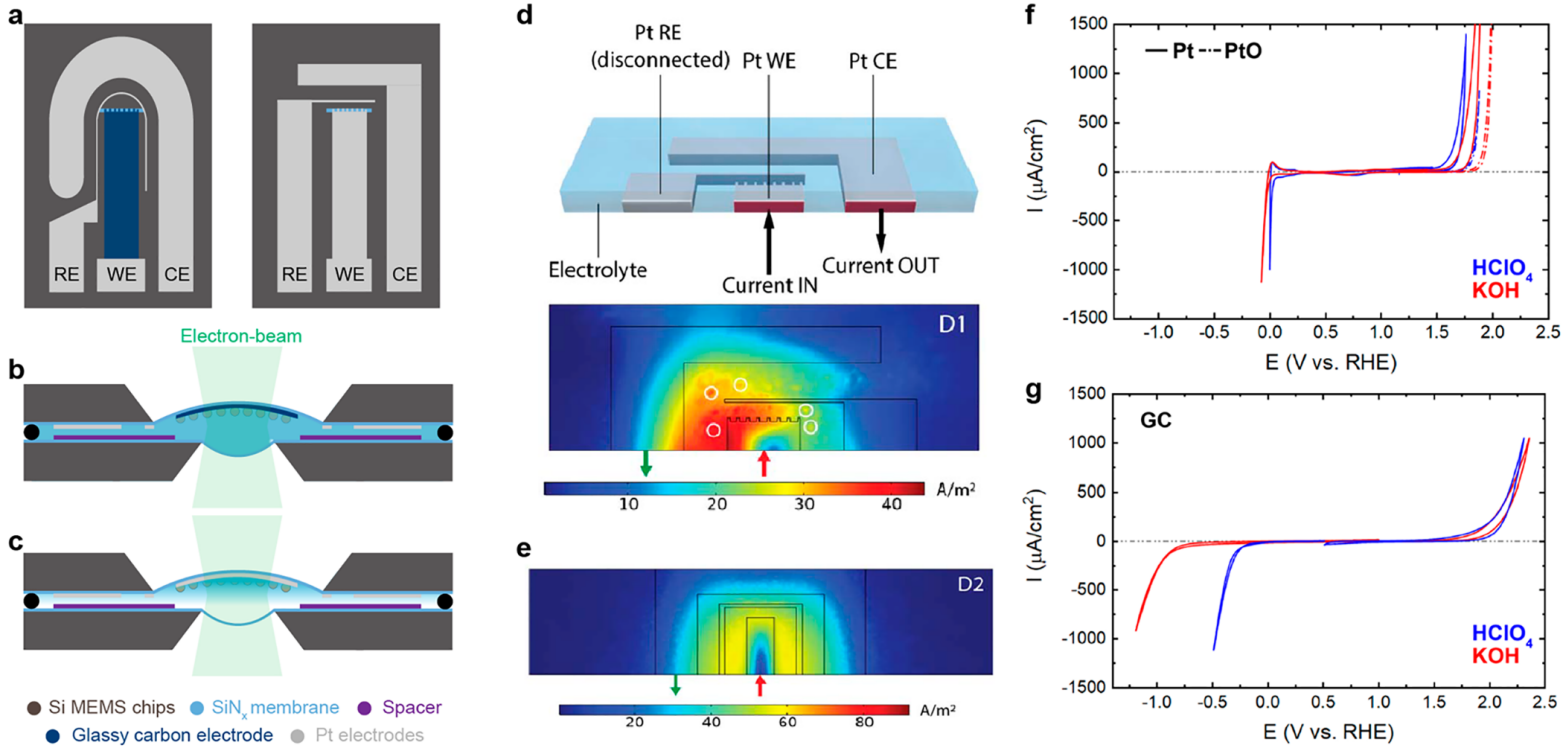
Properties of ec-LPTEM apparatus. (a) Schematics of MEMS-based electrochemical chips where different design and materials of WE, CE, and RE can be fabricated. (b, c) Cross-sectional schematics of the enclosed microcell showing full liquid immersion conditions and wetted thin liquid conditions, respectively. (d) Scheme of FEM simulation geometry and simulated electrolyte current density during OER on nonsymmetrical electrode configuration and (e) on symmetric electrodes. Adapted with permission from ref (15). Copyright 2021 the authors, some rights reserved; exclusive licensee IOP Publishing. Distributed under a Creative Commons Attribution License 4.0 (CC BY) https://creativecommons.org/licenses/by/4.0/. (f, g) Inert potential windows within which Pt and GC electrodes, respectively, are adequate substrates for the study of electrocatalysts. Adapted with permission from ref (1). Copyright 2023 the authors, some rights reserved; exclusive licensee IOP Publishing. Distributed under a Creative Commons Attribution License 4.0 (CC BY) https://creativecommons.org/licenses/by/4.0/.

First, the volume or thickness of the liquid electrolyte is distinctively constrained. The cell can be either completely filled or, alternatively, restricted to a thin wetting film. In the former case, the liquid thickness is determined by the height of the spacer and the additional bulging of the SiN_*x*_ membranes, reaching up to 1 to 2 μm in the thickest part of the cell,^12^ as illustrated in the cross-sectional schematic of Figure 1b. When a thin film wets the surface, the liquid layer is typically induced by capillary forces resulting from surface plasma treatment and possibly modulated by electrowetting upon biasing^2^ (Figure 1c). The coexistence of gas/vapor in equilibrium with the liquid film under wetting conditions makes the deterministic assessment of the liquid thickness challenging.^1^ This limited liquid thickness is a factor of increased ohmic resistance, especially under the thinnest wetting conditions, which can result in a distortion of voltammetry measurements.^13^ Furthermore, the small cross section also reduces diffusional fluxes at the electrode surface, and these can become a limiting factor for the reaction rate.^14^ In addition, when the cell is operated in conjunction with a syringe pump to induce electrolyte flow and replenish chemical species, conditions of mixed diffusion–convection arise.^1,14^ In filled cells, a direct result of this is the observed increase in peak currents measured by cyclic voltammetry (CV) during electrolyte flow.

The second challenge involves the design and location of the electrodes. All three, working, counter, and reference electrodes (WE, CE, and RE, respectively), are patterned on one chip, that is, in a coplanar geometry (Figure 1a) and in close proximity to each other. Consequently, heterogeneities in the current distribution can arise (Figure 1d), which would directly translate into degradation rates during experiments that are dependent on the position along the electron transparent area. In contrast, a symmetric configuration with respect to the WE and the CE geometry results in a more homogeneous current density distribution as it was calculated using finite element model (FEM) simulations for the case of the kinetically driven OER (Figure 1e).^15^

Third, the electrochemical chips are fabricated using microelectromechanical (MEMS) techniques, and this restricts the choice of materials. The substrate and reference electrodes are critical parameters and can drastically modify the results and interpretation of electrocatalytic experiments.^16,17^ We have shown that Pt electrodes could be adequate substrates for oxygen-evolving catalysts, especially when passivated with an oxide layer induced by plasma treatment (Figure 1f).^30,31^ However, for reactions where Pt as a substrate exhibits high activity such as the ORR or induces competitive reactions in the potential range of interest such as the CO_2_ER, an inert yet conductive substrate is required. In this case, amorphous, glassy carbon (GC) electrodes have been shown to be excellent candidates. When we tested the electrochemically inert window for customized or commercial GC electrodes, we found them to be an adequate choice for many electrocatalysts studies, including the CO_2_ER at low potential, the ORR, and the OER, and under alkaline, neutral, or acidic conditions (Figure 1g).^1,14^ We note that these electrodes show a typically increased resistivity (and hence a higher ohmic drop) and that owing to the difficulties of pretreating GC electrodes in the ec-LPTEM apparatus, there are often increased resistances that are attributed to slow charge-transfer rates at the electrolyte–electrode interface.^3,14^ Additionally, we have found them to be highly fragile and they can withstand only limited reaction times in the highly negative potential ranges.^14^

Finally, MEMS techniques also impose restrictions on the patterning of the reference electrode. True REs, where a single and well-defined redox-couple sets the reference potential, have not been miniaturized to the scale of the microcells and potential measurements continue to be performed with metallic quasi-reference electrodes. As a result, the reference potential is ill-defined, requires calibration, and may be subject to strong drifts.^16,18^ Calibration is typically done by using a reference electrochemical system,^14^ another true reference in a dedicated ex situ cell,^3^ or by using specific features of the system under study, for instance, the Pt–O reduction peak or hydrogen under potential deposition (HUPD) features on Pt.^1^ As an alternative, recent developments have seen true REs placed upstream, in a dedicated cavity within the shaft of the holder^1,19^ or used within the framework of closed bipolar electrodes^20^ where a connector output from the cell is used as an external working electrode at which potentials can be measured outside the holder; however, matching electrolytes are required on both sides of the connector, which can be a challenge.^20^

Taking into account the system’s constraints, such as the ones described herein, it becomes obvious that the ec-LPTEM setup needs to be uniquely tailored for each electrocatalytic or electrochemical process of interest with customized options for yielding realistic reactions within its microcells. Next, we describe valuable mechanistic insights into our understanding of electrocatalytic transformations elucidated by real-time ec-LPTEM observations designed and performed in our laboratory.

## OER Catalysts

Oxygen electrocatalysis, including OER and ORR, is governed by high overpotential values for driving the reactions, and it arises as the bottleneck for efficient electrocatalytic devices.^21^ More specifically, alkaline OER can produce green hydrogen gas via water electrolysis technologies. Research on electrocatalysts that can lower the kinetic barrier of the OER is ongoing. To date, oxides with diverse crystal structures and electronic properties have been shown to be highly suitable as OER catalysts, but questions pertaining to the prevailing mechanism of operation remain open.^22,23^ Understanding their stability and/or transformations under operational conditions mainly involves monitoring surface redox reactions and morphological electrocatalytically induced changes. Thus, many operando characterization methodologies have been implemented for OER studies, with the majority of them involving X-ray-based techniques, while very few reports have surfaced that use ec-LPTEM.^24^ Ortiz-Pena et al.^25^ recently performed in situ TEM measurements of Co_3_O_4_ nanoparticles under OER conditions in a neutral phosphate electrolyte, and they showed an irreversible phase transformation of the particles to an amorphous phase when applying constant current. They reported that crystalline Co_3_O_4_ nanoparticles were embedded in a matrix of the amorphous Co oxyhydroxide-like phase; however, the formation of the highly oxyhydroxide phase is a reversible process, to an extent that it is difficult to stabilize for its observation, in real time or post-mortem.

**Figure 2:**
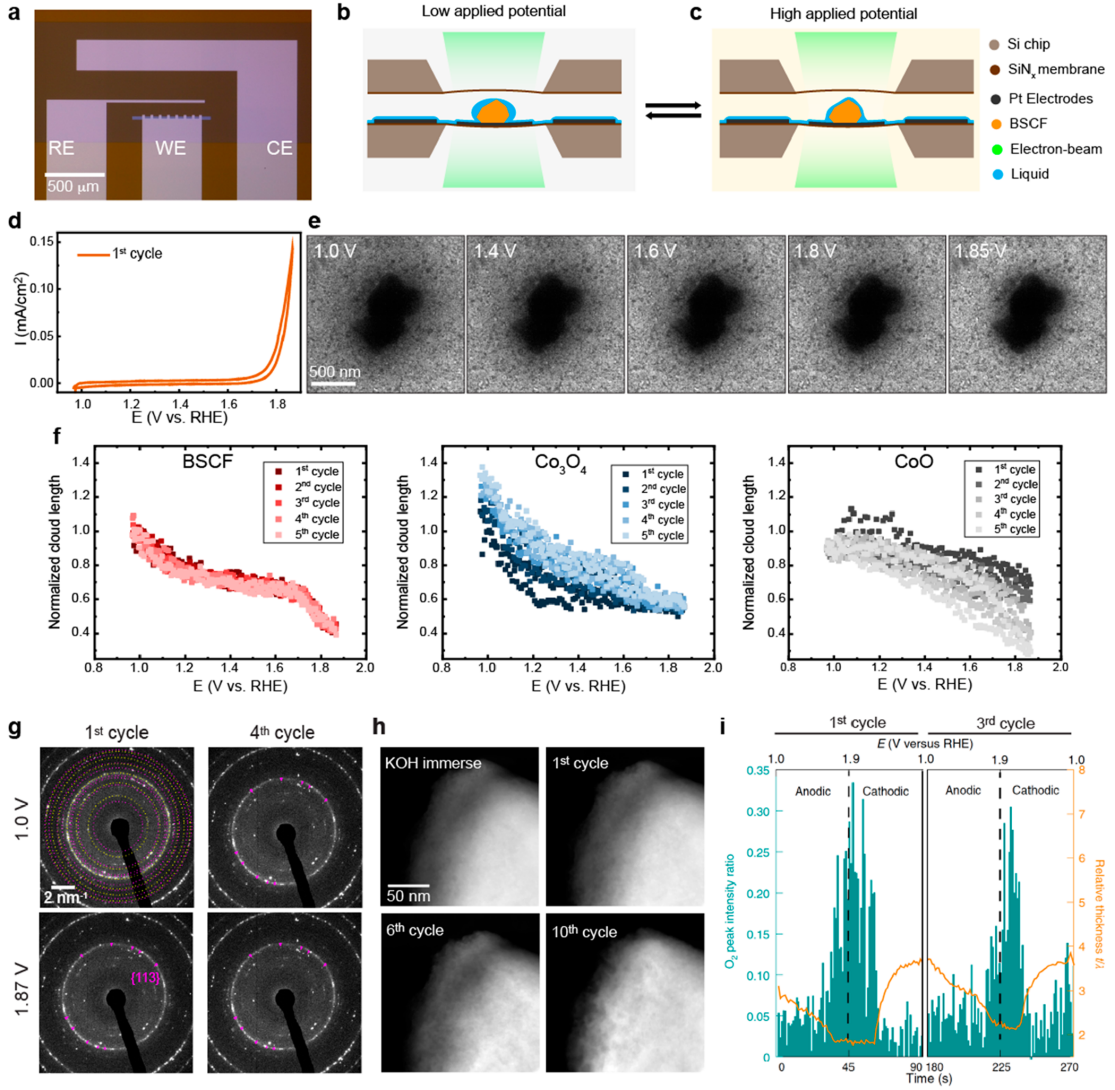
OER studies of Co-based oxide catalysts in ec-LPTEM. (a) Optical microscopy image of MEMS electrochemical chip with three Pt thin-film electrodes. (b, c) Schematics of reversible liquid movement surrounding an oxide particle in a liquid-cell enclosure at low and high potential, respectively. (d) CV of first cycle and (e) TEM images of BSCF particle at different potential stages for the first cycle. (f) Normalized cloud length as a function of applied potential of three Co oxides. (g) SAED patterns for the first and fourth cycles at 1.0 and 1.87 V vs RHE. Yellow and magenta rings indicate BSCF perovskite and Co/Fe spinel reflections, respectively. The magenta arrows indicate {113} reflections of the Co/Fe spinel. (h) Identical location imaging of a BSCF particle at various cycles. (i) O_2_ peak intensity ratio (green) and EELS-calculated relative thickness (orange curve) with respect to elapsed time (bottom axis) and applied potential (top axis). a–g and i were adapted with permission from ref (2). Copyright 2023 the authors, some rights reserved; exclusive licensee Springer Nature. Distributed under a Creative Commons Attribution License 4.0 (CC BY) https://creativecommons.org/licenses/by/4.0/. h was adapted with permission from ref (26). Copyright 2020 the authors, some rights reserved; exclusive licensee American Chemical Society. Distributed under a Creative Commons Attribution License 4.0 (CC BY) https://creativecommons.org/licenses/by/4.0/.

To evaluate the extent of these transformations under realistic OER conditions, we performed ec-LPTEM measurements of Co-based oxide catalysts in alkaline electrolytes during potential cycling.^2^ We used an all-Pt electrode electrochemical chip to drop cast the catalyst (Figure 2a). To resolve the particles and their changes in transmission mode with a low electron dose, we configured the system to allow for a thin wetting layer on the electrodes (Figure 2b). Sequences of TEM images (Figure 2e) were acquired with high temporal resolution during several cycles of electrochemical CV measurements in the OER range of [1.0–1.87] V vs the reversible hydrogen electrode (RHE) (Figure 2d). The potential-dependent variation of the local contrast was associated with the modification of the wettability at the oxide surfaces, as illustrated in Figure 2c. The wetting behavior around the particle was extracted by probing the alteration of lateral liquid thickness around the particle at different potentials. A comparison of the highly OER-active perovskite Ba_0.5_Sr_0.5_Co_0.8_Fe_0.2_O_3-δ_ (BSCF) to the spinel Co_3_O_4_ and the rock salt CoO revealed interesting wetting properties of the different structures with respect to the active Co site (Figure 2f). In all cases, at low applied potential, the hydrophobic surface character of the oxide surfaces attracted liquid, and at high applied potential, a more hydrophilic surface character was probed and attributed to electrowetting induced by OH^–^ accumulation at the interfaces. As seen in the plots in Figure 2f, BSCF and Co_3_O_4_ exhibited a distinct transition toward hydrophilicity at ∼1.2 V vs RHE, which was associated with the redox Co^2+^/Co^3+^ reaction and which alters the interfacial capacitance. This was related to the formation of the Co-oxyhydroxide phase in both oxides, and it suggested the presence of Co^2+^ in the tetrahedral site at the surfaces of BSCF. Remarkably, the BSCF particle did not exhibit any observable changes and was found to be stable during cycling. By performing operando selected-area electron diffraction (SAED) (Figure 2g), we also showed that the perovskite BSCF particles were composed of a Co/Fe spinel surface with cobalt(II) valence, and during cycling, the subtle changes in the {113} spinel reflections indicated restructuring of the spinel surface (to the oxyhydroxide phase). The stability of the BSCF and the reversibility of the structural processes during cycling were further supported by identical location studies,^26^ with the only visible effect being a tendency of the BSCF surface to become porous, with increasing porosity observed as the number of cycles increased (Figure 2h). The hypothesis of BSCF’s porous surface after CV has been proposed previously;^27^ however, we note that this degradation could also be induced by electron beam irradiation and handling of the sample ex situ, and may not be inherent to the reaction processes. Finally, further operando measurements using electron energy loss spectroscopy (EELS) provided direct evidence of the formation of molecular oxygen (O_2_) during cycling (Figure 2i). The O_2_ peak intensity ratio in EELS evolved periodically as a function of applied potential, and we were able to link the O_2_ evolution to the change in the cloud length at ∼1.65 V versus RHE in Figure 2f, which indicated the further consumption of the liquid electrolyte around the particle during OER. Encouraged by the discovery that we can site-specifically probe the reaction products, we have embarked on further experiments for obtaining quantitative information on the concentration of the product of oxygen electrocatalysts as probed by EELS inside the liquid-containing TEM microcells.^11^

In conclusion, our observations using ec-LTEM for OER catalysts provided evidence of potential-regulated switchable wetting in oxides by probing effects that include electrowetting, surface redox reactions, structural changes, and product formation. This work exemplifies the entirely novel insights we can strive to obtain with the surface-sensitive ec-LPTEM techniques and their value in aiding developments toward targeted catalyst design.

## ORR Catalysts and Catalyst Layers

The slow reaction at the cathode side of proton exchange membrane fuel cells (PEMFC) that limits the performance of these devices for use in a variety of power applications is the ORR.^28^ To accelerate its kinetics and achieve maximum power density, several strategies on the design of the catalyst layers, which consist of a heterogeneous network of metallic Pt nanoparticles on carbon supports covered by a thin layer of ionomer, are pursued. The reduction of Pt loading is an unchanging goal and typically involves increasing the catalysts’ mass activity with Pt-M alloys (where M is a transition metal, typically Fe, Co, Ni, or Cu) of tuned composition and/or controlled shapes to preferentially expose the most active facets.^29^ Alternatively, modifications of the other components, the carbon supports, or the ionomer network can also improve Pt utilization.^29^ In either case, issues with the durability of the cells have not yet been surpassed. These have to do with the reduction of the electrochemical surface area or specific activity over time and are linked to metal dissolution and dealloying, coarsening or detachment of nanoparticles, and shape changes during operation.^30^ Toward the understanding of these transformation-based performance losses, ec-LPTEM studies of electrocatalysts for the ORR typically seek to reproduce accelerated stress tests (ASTs) in which catalysts are cycled repeatedly through Pt oxidation and reduction potentials to mimic potential variations during operation and start-up/shut-down events, up to a range as wide as [0.0, 1.45] V vs RHE. Previous studies using ec-LPTEM utilized carbon-supported Pt-alloyed nanoparticles of a nominal size of 8 nm, and various processes such as potential-dependent material dissolution redeposition and particle displacement at the surface of the supports resulting in coalescence events were reported.^31,32^

**Figure 3:**
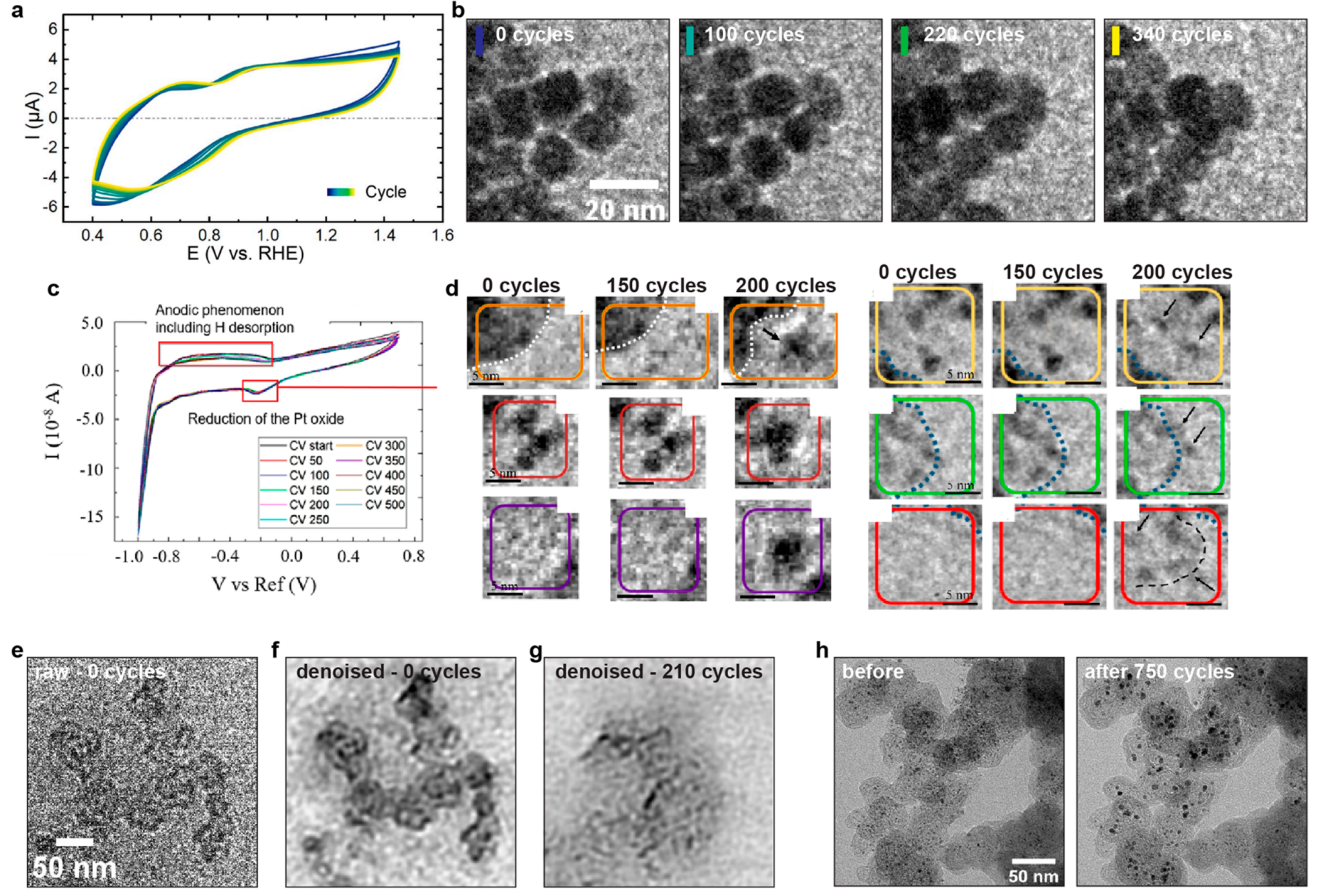
ORR studies of Pt nanocatalysts in ec-LPTEM. (a) CVs of 8 nm Pt nanocubes and (b) representative sequences of TEM images. Adapted with permission from ref (1). Copyright 2023 the authors, some rights reserved; exclusive licensee IOP Publishing. Distributed under a Creative Commons Attribution License 4.0 (CC BY) https://creativecommons.org/licenses/by/4.0/. (c) CVs referenced to Pt RE and (d) representative transformations of the Pt/C/Nafion catalyst layer captured in TEM. Reproduced with permission from ref (33). Copyright 2020 American Chemical Society. (e) Raw and (f) denoised TEM images of a Pt/Ketjenblack aggregate and (g) its damage after cycling in ec-LPTEM. (h) Identical location studies of the same catalyst as that in (e).

In our laboratory, we began by looking into the transformations of 8 nm pure Pt nanocubes using electrochemical conditions closely mimicking ASTs.^1^ After stabilizing the nanoparticles at a low electron dose, we performed cyclic voltammetry in the range of [0.4, 1.45] V vs RHE in 0.1 M HClO_4_. The subtle oxidation and reduction features in the CVs of Figure 3a for the duration of the experiment (500 cycles) pointed toward minor changes in the active surface area. Still, Pt dissolution was observed, with particles shrinking while becoming increasingly rounded and possibly merging into each other (Figure 3b). The relative area loss of Pt after 500 cycles was calculated to be around 10%, similar to that of the particles located in the nonelectron-beam-irradiated area, which is statistically significant for the overall loss in activity of the catalysts. In the study of conventional Pt/C catalysts, however, the individual Pt nanoparticles are on the order of 2 to 3 nm in diameter, and their changes are more difficult to resolve with ec-LPTEM. Impagnatiello et al.^33^ used a conventional Pt/C catalyst imbedded in an ionomer matrix and deposited on the working electrode prior to filling the microcell with the acidic electrolyte. By performing CV measurements for 500 cycles (referenced to the Pt electrode, Figure 3c), they showed a wide variety of processes occurring across the sample, including detachment, coalescence, dissolution, and reprecipitation, all under similar cycling conditions (Figure 3d). Even though the authors argued that they minimized direct electron-beam-induced damage to the metallic specimen, it is expected that real-time ec-LPTEM will be increasingly volatile for the conventional Pt/C/ionomer system since the carbon supports are more readily prone to corrosion and ionomers are difficult to stabilize in ambient conditions used for in-situ TEM measurements. Thus, in an effort to decouple the electrochemically induced degradation mechanisms for conventional catalyst layers, we applied the electrochemical conditions as in the case of the Pt nanocubes^1^ on 2 to 3 nm Pt nanoparticles on Ketjenblack porous carbon supports.^34^ The electron dose was considerably reduced to mitigate radiation damage, which limited the achievable resolution (Figure 3e). Despite improvements from deep-learning-based denoising algorithms, individual Pt nanoparticles were not resolved^34^ (Figure 3f). Furthermore, we observed substantial changes to the carbons after in situ cycling (Figure 3g). This was in stark contrast to similar conditions applied for identical location studies where, instead, only Pt coarsening was observed^34^ (Figure 3h). Thus, comparison with identical location imaging or post-mortem experiments are necessary to understand the interactions with the electron beam that can modify the degradation pathways.

## CO_2_ Electroreduction Catalysts

CO_2_ER holds the promise of a closed-loop carbon cycle, and assessing the Faradaic efficiency, selectivity, and stability of various catalysts toward the multicarbon products is a critical interest for the energy community.^35,36^ Among numerous, mainly metallic, materials that have been studied, Cu’s ability to form beyond CO products is remarkable.^37^ Cu can further be tuned to selectively catalyze ethanol, ethylene, and in general C_2+_ products, as needed.^38^ However, despite the plethora of active research, Cu’s efficiency and selectivity are hindered by the stability of the nanoscale polymorphs during operation.^39^ Whereas the efficiency and selectivity of the catalysts are well determined by electrochemical methods, stability- and degradation-related mechanistic studies require real-time observations. Early on, the need for operando characterization of Cu nanocatalysts at CO_2_ reducing potentials led researchers to develop the techniques to mimic, as much as possible, realistic conditions.^40^ The development of ec-LPTEM with respect to its application to CO_2_ electroreduction was rather delayed due to the technical challenges associated with the spatial resolution in liquids for nano-objects and, importantly, due to the negative potentials needed to trigger the reaction and its possible hindrance by the competing hydrogen evolution reaction. Thus, most studies on Cu for CO_2_ER demonstrated the growth of particles^41^ or remained at low operating potentials,^42,43^ owing to the stability of the commercially available substrates of choice.

**Figure 4:**
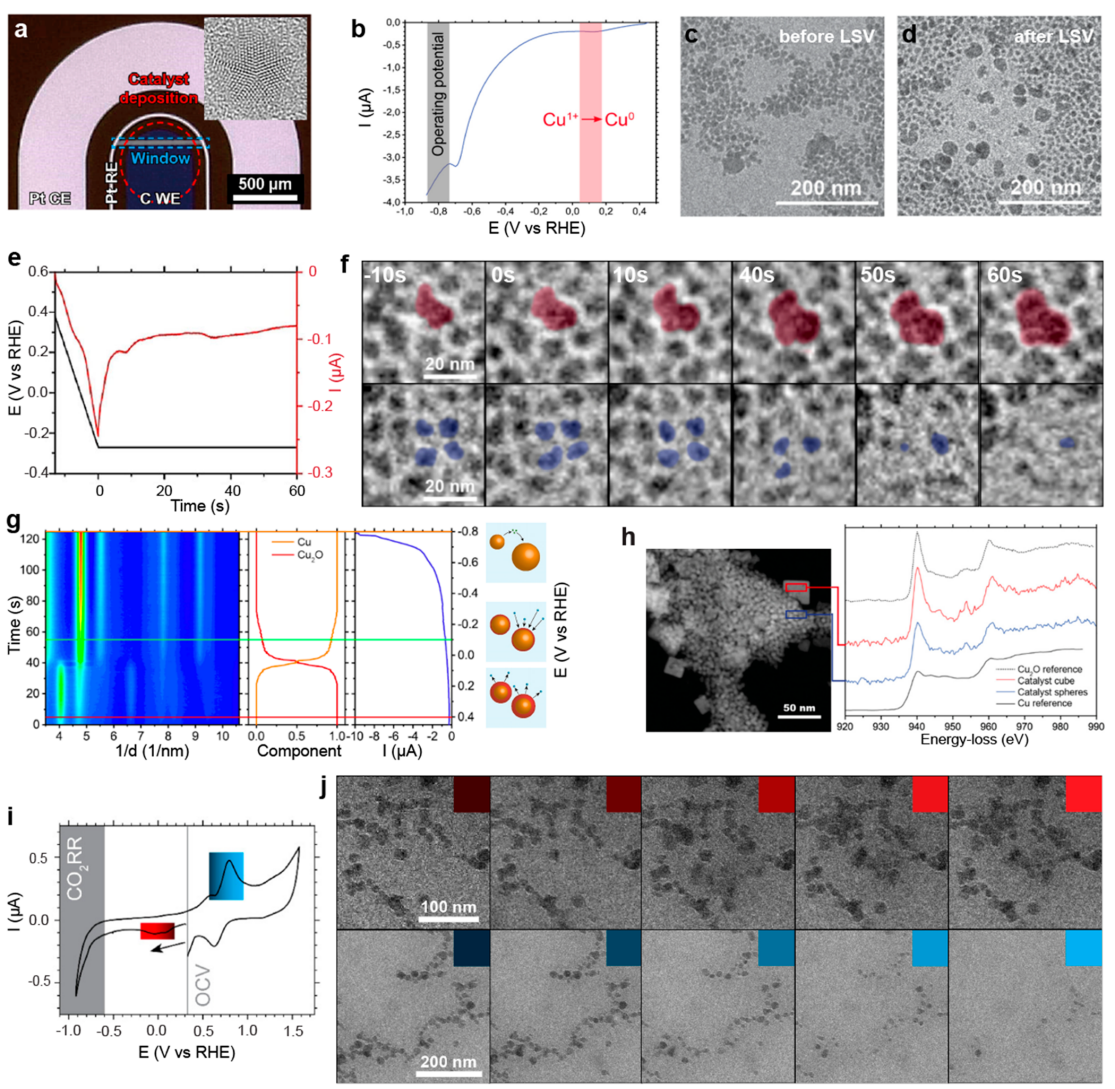
CO_2_ER studies of Cu nanospheres in ec-LPTEM. (a) Optical microscopy image of a custom-fabricated GC chip showing the design of the three electrodes and (inset) high-resolution TEM image of a 7 nm Cu nanosphere. (b) Current response in LSV from positive to −0.9 V vs RHE and TEM images of the spheres before (c) and after (d) the LSV measurement. (e) LSV followed by CA and its response followed by the evolution of the Cu nanoparticles (f). (g) Operando SAED from the OCV to −0.8 V and schematics of the evolution of the particles. (h) Post-mortem scanning TEM image of the agglomerated Cu nano-objects and EEL spectra of the Cu L_3,2_ edge. Adapted with permission from ref (3). Copyright 2020 Wiley-VCH GmbH. (i) CV measurement of the LSV/CA structures and (j) evolution of the secondary particles during cathodic (red) and anodic (blue) potential. Adapted with permission from ref (1). Copyright 2023 the authors, some rights reserved; exclusive licensee IOP Publishing. Distributed under a Creative Commons Attribution License 4.0 (CC BY) https://creativecommons.org/licenses/by/4.0/.

Considering the critical role of the substrate electrode toward reliable CO_2_ER-related measurements, we first designed a three-electrode electrochemical chip and fabricated a customized GC WE (Figure 4a) to expand its inert range toward negative potentials.^3^ A stable operating window could be operated up to −0.8 V vs RHE (Figure 4b) for about 60 s, prior to mechanical failure of the system. Using this system, we performed the first breakthrough CO_2_ER study,^3^ using ec-LPTEM exclusively, on 7 nm Cu nanospheres (inset of Figure 4a). In general, the majority of in situ TEM studies are centered around Cu nanospheres rather than other shapes because, despite their nonspecific selectivity, their rapid transformation during operation allows their real-time tracking within the short operating time window of ec-LPTEM measurements. This transformation mainly involves the evolution of the nanospheres to secondary particles, as depicted in the TEM ex situ images before and after linear sweep voltammetry (LSV) measurements (Figure 4c,d). The exact pathway of the dissolution/redeposition process in these early steps of the CO_2_ER had not been determined in the past. To do so, we designed an in situ electrochemical protocol that involved three steps; starting at open circuit potential (OCV), a short interval of LSV was then applied prior to the final step of maintaining a constant potential (chronoamperometry, CA), (Figure 4e). The operando ec-LPTEM observations unambiguously revealed that the primary-to-secondary particle growth proceeds solely through Ostwald ripening without coalescence events taking place. To complement the morphological monitoring, operando SAED was used in a similar experiment to determine the structural changes for these first stages of CO_2_ER^1^ (Figure 4g). It is well known that Cu oxidizes quickly, and its metallic, as-synthesized state is difficult, if not impossible, to attain at the start, even in bulk cell experiments. The fingerprint peak at the LSV response in Figure 4b already suggested a Cu^1+^ to Cu^0^ transformation at about 0 V vs RHE, but the full dynamic evolution of the structure, as depicted in Figure 4g, facilitated mechanistic insights into the electrochemistry driving the activation process of Cu nanospheres. Specifically, we argued that the nanospheres first oxidize upon immersion in the neutral electrolyte where Cu_2_O, Cu^1+^, and Cu^2+^ species coexist, possibly due to the large surface-to-volume ratio of the spherical shape. Then during cell startup, the applied cathodic potential results in the growth of metallic Cu secondary particles and subsequent reduction of the oxidized shells of the primary particles. Finally, during operation at constant potential and by means of transient Cu species, further growth of secondary particles is achieved by Ostwald ripening. We note that conversion of the secondary particles to a cubic shape was not observed in situ, and it is generally accepted that their presence in post-mortem studies in oxidized form (Figure 4h) is a result of their air exposure, highlighting the susceptibility of redeposited Cu to oxidize. A subsequent ec-LPTEM study involving the activated Cu particles was designed to mimic the start/stop operation of CO_2_ER. This time, cyclic voltammetry was used, starting from OCV toward a negative potential of −1.0 V vs RHE, ending back at OCV through positive, oxidizing potentials (Figure 4i). Redeposition of the dissolved Cu_2_O, Cu^1+^, and Cu^2+^ species starting at OCV was identified for the extent of the reduction feature (∼0 V vs RHE) in the voltammogram and significant dissolution at the oxidative peak (∼0.8 V vs RHE) (Figure 4j) was recorded. Interestingly, no morphological changes were observed between the reduction peak and the more relevant cathodic potential of −1.0 V vs RHE, and no redeposition from the oxidative peak on the return to the OCV was resolved, despite the additional reduction features.

In conclusion, advancements of the confined TEM microcells for enduring negative potentials have allowed us to study CO_2_ER processes in real time with remarkable results on the dissolution/redeposition pathways. Thus, the unique insights of ec-LPTEM experiments can motivate descriptors relating the electrochemical stimuli to the stability for various nanocatalysts.

## Conclusions and Outlook

Our aspiration with this Account was to demonstrate the breadth of information that can be uniquely acquired and interpreted toward the mechanistic understanding of electrocatalyst transformations with ec-LPTEM. We discussed the approach of our laboratory and, focusing on the scientific outcomes, showed that the technique has matured beyond the typical morphological imaging with insights ranging from the electrowetting behavior of oxide nanocatalysts to the restructuring features of metallic nanocatalysts. We showed that the chemical surroundings of single particles undergoing electrocatalytic reactions can be probed in real time using EELS, and we foresee that apart from the exceptional results of ec-LPTEM on the stability mechanisms of electrocatalysts, selectivity studies, through either quantitative EELS or mass spectroscopy measurements, could be achieved. In recent years, remarkable advancements in camera technologies for the collection of electron signals have allowed reasonable dissipation of the effects of electron beam irradiation in liquids while enhancing temporal resolution. Critically, spatial resolution during electrochemical measurements in liquids remains a challenge. We foresee that future developments in electrode design and materials will improve visualization and perhaps realize atomic resolution imaging of the transformation processes. Finally, we should not overlook the fact that electrocatalytic reactions occur over a range of length and time scales. In the future, linking the atomic-to-microscale transformations and assessing these effects for the long-term stability or aging of catalysts and catalyst layers will be critical. How the fundamental processes probed by ec-LPTEM affect the practical considerations of the operation of electrocatalytic devices remains to be addressed; therefore, the close collaboration of all stakeholders can aid in the pursuit of solutions for the pressing global issues related to the performance of electrochemical systems.
